# Cluster analysis driven by unsupervised latent feature learning of medications to identify novel pharmacophenotypes of critically ill patients

**DOI:** 10.1038/s41598-023-42657-2

**Published:** 2023-09-20

**Authors:** Andrea Sikora, Hayoung Jeong, Mengyun Yu, Xianyan Chen, Brian Murray, Rishikesan Kamaleswaran

**Affiliations:** 1 Department of Clinical and Administrative Pharmacy, University of Georgia College of Pharmacy, Augusta, GA USA; 2 Georgia Institute of Technology, Atlanta, GA USA; 3 Department of Statistics, University of Georgia Franklin College of Arts and Sciences, Athens, USA; 4 Department of Pharmacy, University of North Carolina Medical Center, Chapel Hill, NC USA; 5 Department of Biomedical Informatics, Emory University School of Medicine, Atlanta, GA USA; 6 Department of Biomedical Engineering, Georgia Institute of Technology, Atlanta, GA USA

**Keywords:** Outcomes research, Translational research

## Abstract

Unsupervised clustering of intensive care unit (ICU) medications may identify unique medication clusters (i.e., pharmacophenotypes) in critically ill adults. We performed an unsupervised analysis with Restricted Boltzmann Machine of 991 medications profiles of patients managed in the ICU to explore pharmacophenotypes that correlated with ICU complications (e.g., mechanical ventilation) and patient-centered outcomes (e.g., length of stay, mortality). Six unique pharmacophenotypes were observed, with unique medication profiles and clinically relevant differences in ICU complications and patient-centered outcomes. While pharmacophenotypes 2 and 4 had no statistically significant difference in ICU length of stay, duration of mechanical ventilation, or duration of vasopressor use, their mortality differed significantly (9.0% vs. 21.9%, p < 0.0001). Pharmacophenotype 4 had a mortality rate of 21.9%, compared with the rest of the pharmacophenotypes ranging from 2.5 to 9%. Phenotyping approaches have shown promise in classifying the heterogenous syndromes of critical illness to predict treatment response and guide clinical decision support systems but have never included comprehensive medication information. This first-ever machine learning approach revealed differences among empirically-derived subgroups of ICU patients that are not typically revealed by traditional classifiers. Identification of pharmacophenotypes may enable enhanced decision making to optimize treatment decisions.

## Introduction

Medication regimens of critically ill patients in the intensive care unit (ICU) are complex and heterogeneous^1,2^. This heterogeneity of medication regimens has parallels to the common and lethal disease states of critical illness including sepsis and acute respiratory distress syndrome (ARDS)^3,4^. Managing the heterogeneity of critical illness is a nearly universally cited challenge for ICU clinicians and researchers^5,6^. Phenotyping has been proposed to identify patterns of diagnosis and treatment response among these complex heterogenous syndromes^7–9^. In particular, phenotyping via artificial intelligence (AI) and machine learning (ML) has demonstrated potential to be a powerful methodology to handle Big Data generated by critically ill patients for identification of novel patient subgroups and prediction of patient outcomes including sepsis, acute kidney injury, mechanical ventilation, ARDS, and more^10–17^. However, to date, this methodology has only been applied in a limited fashion to the highly complex and heterogenous nature of ICU medication regimens^1^.

Critically ill patients are often prescribed greater than 20 medications, with many deemed high-risk for patient harm by the Institute of Safe Medication Practices^18–21^. Further, it has been estimated that each day, a critically ill patient will suffer at least one medication related error. These medication related errors can lead to serious adverse drug events associated with a doubled risk of mortality^20,21^. Medication therapy optimization has significant potential to improve patient outcomes and reduce healthcare costs^1,2^. Thus, the development of novel prediction models with granular medication information to predict adverse events and direct resources is warranted^1^. However, identifying patterns associating medication therapy with patient outcomes within the vast amounts of data generated by ICU patients has remained a challenge, and to date, no AI/ML models have incorporated comprehensive ICU medication regimens into their analyses^22^.

We hypothesized that a similar approach as has been explored with other disease states of critical illness could be applied to ICU medications. Here, we sought to identify novel pharmacophenotypes using unsupervised machine learning to cluster medications used in the ICU and explore their relationship to patient-centered outcomes.

## Methods

### Study sample

Patients were drawn from the University of North Carolina Health System, an integrated healthcare delivery system where clinical care is managed via a comprehensive electronic health record (EHR). Patients were included if they were ≥ 18 years old with an ICU admission greater than 24 h between October 2015 and October 2020. ICUs included medical, surgical, neurosciences, and burn specialties. The hospitals varied including community hospitals and academic medical centers. Only the index ICU admission per each patient was considered in this analysis. The institutional review board at The University of Georgia approved this study and included waiver of consent (PROJECT00002652), and all methods were performed in accordance with the relevant guidelines and regulations.

The EHR was queried for patient demographics, medication information, and patient outcomes. Patient demographics included age, sex, admission diagnosis, ICU type, and Acute Physiology and Chronic Health Evaluation II. Medication information including drug, dose, route, duration, and timing of administration were recorded. Patient outcomes included mortality, hospital length of stay, development of delirium (defined by a CAM-ICU positive score), duration of mechanical ventilation, duration of vasopressor use, and acute kidney injury (defined by the presence of renal replacement therapy or a serum creatinine greater than 1.5 × baseline).

### Feature extraction

#### Patient demographics

There were 30,550 given medication entries in the dataset from a total of 991 patients. Of these 30,550 administered medications, there were 440 unique medications when the filter of generic drug names was used and when dose and route information were excluded (e.g., cefepime 1gm and 2gm were counted under the feature of cefepime). Medication records from the raw dataset included a variety of medication administration record (MAR) actions including “given”, “missed”, “hold,” etc. To ensure this analysis only included records of medication that were administered to the patient (not just ordered), only the entries where the medication action label corresponded to "Given", "New Bag", "Restarted," or "Rate Change" were used for the analysis. Some entries contained "free-text" for ICU personnel communication purposes and were discarded. Additionally, duplicate and incomplete entries were filtered out. After cleaning the dataset, the data were transformed into a binary (boolean) vectored form where the 440 unique medications were assigned as the rows, and 991 patients were assigned as the columns. For each patient, a binary value of 1 was assigned to indicate whether the patient received a particular drug. For patient outcomes, the labels for categorical features were relabeled as numeric values. In the cases of unknown or missing entities, these were replaced with “negative” or “no.” The entire mapping of original labels to new labels is provided in Appendix Table 1.

### Unsupervised learning approach

#### Medication clustering

After performing principal component analysis (PCA) on the large, binary medication dataset, the Restricted Boltzmann Machine (RBM) was used to further enrich the latent feature space, which was use as input to the hierarchical clustering algorithm to support the novel discovery of unique pharmacotherapy profiles^23^.

#### Principal component analysis

During PCA, each of the 440 unique medications was treated as an independent variable. PCA is a widely used dimensionality reduction technique to reduce the dimensionality of a dataset with p random variables to q, which is the desired number of variables^24^. The optimal number of principal components was selected after plotting the explained variance against the number of principal components (see Appendix Fig. 1). The number of principal components was selected as 150 to maintain sufficient variance (approximately 75%) in the data while significantly reducing the dimensionality.

#### Restricted Boltzmann Machine

**Figure 1:**
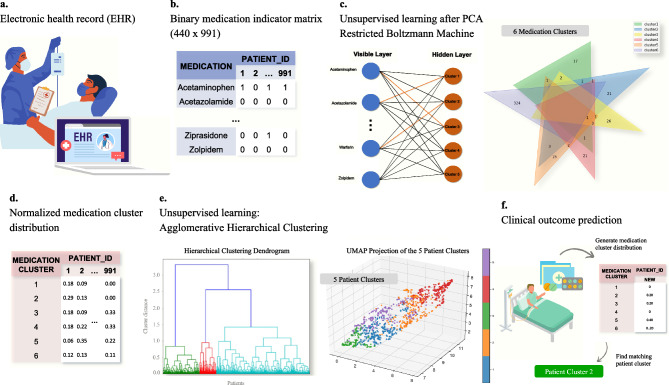
Pharmacophenotype derivation workflow. (**a**) When medications are ordered by the clinician for ICU patients, all administered medications are recorded and stored in the electronic health record (EHR) system. (**b**) The medication data from the EHR was preprocessed to create a binary indicator matrix that contains all unique medications taken by a total of 991 patients. (**c**) Five medication clusters were created using unsupervised learning model (Restricted Boltzmann Machine). The layers that are not turned “on” (indicated in orange) to any hidden layers are grouped as an extra sixth cluster. (**d**) For each patient, the frequency of each medication cluster was counted and normalized by the total medications taken by each patient during their stay. (**e**) The normalized medication cluster distribution of each patient is used as a feature to agglomerative hierarchical clustering to develop novel pharmacophenotypes of critically ill patients. (**f**) These novel pharmacophenotypes can be used to predict clinical outcomes of new patients based on their medication regimens.

RBM was used to learn unsupervised feature abstractions or ‘latent factors’ of the PCA reduced data^25^. RBM is a simple, two-layered neural network with one visible layer and one hidden layer. It is typically used for collaborative filtering as RBM is capable of learning internal representations of the input variables using unsupervised methods enabling complex relationships to be discovered in the process. For medication clustering purposes, we trained the RBM^25,26^ to learn the high dimensional and non-linear nature among medication assignments based on the co-occurrence of medications for each patient. The default hyperparameters for implementation were used based on the works of Chen^26^. From each patient’s binary assignment of medications, the RBM learned the weight coefficients to ultimately determine which nodes out of all nodes were activated or inactivated for each hidden unit. For clustering purposes, each medication is an independent node from the visible layer (440 units), and connections that are activated to a single hidden layer indicate cluster assignment (see Fig. 1). For example, if acetaminophen (from the visible layer) and Cluster 1 (from the hidden layer) connection was activated, acetaminophen would be assigned to Cluster 1. After assigning medications to each cluster from the created hidden layers, medications that were unassigned (never activated in the five hidden layers) were grouped as Cluster 6. Table 1 lists the medications assigned to Clusters 1–5, and Table 2 lists the unassigned medications in Cluster 6.

**Table 1:** Medication clusters assigned by restricted boltzman machine.

Cluster 1	Cluster 2	Cluster 3	Cluster 4	Cluster 5
Amitriptyline Atorvastatin Biotin Buprenorphine Cefazolin Cefuroxime Chlorothiazide Cholecalciferol Clindamycin Emtricitabine-Tenofovir Ergocalciferol Melatonin Metolazone Osimertinib Oxybutynin Pantoprazole Potassium/Sodium phosphates Sennosides Silver sulfadiazine Thrombin	Alteplase Atovaquone Barium sulfate Basiliximab infusion Bumetanide Cefuroxime Citalopram Cyclosporine Dextrose Docusate sodium Dutasteride Fentanyl Ferrous sulfate Gentamicin Glucose Hydrocortisone Hydroxychloroquine Hydroxyurea Lopinavir-ritonavir Methocarbamol Midodrine Oxcarbazepine Pentamidine Simvastatin Ticagrelor Ursodiol	Adenosine Amiodarone Ampicillin Anakinra Biotin Bivalirudin Cefazolin Cefdinir Cetirizine Clobazam Dopamine Droxidopa Esomeprazole Magnesium Estradiol Ganciclovir Indomethacin Mirtazapine Moxifloxacin Multivitamin Nicardipine Olanzapine Oxycodone-acetaminophen Racepinephrine Rivaroxaban Sodium acetate Sodium chloride Sumatriptan Tamsulosin Trazodone Triamcinolone Venlafaxine	Aluminum-mag hydroxide-simethicone Amitriptyline Amphotericin B Liposomal Amphotericin B Aspirin Azelastine Bupivacaine Buspirone Calcium carbonate Carbidopa Citrate dextrose Codeine Conjugated-estrogens Daunorubicin Hydroxychloroquine Lactobacillus Mafenide Metformin Montelukast Neomycin Nicardipine Nifedipine Peramivir Polyethylene glycol Potassium citrate Pravastatin Sodium chloride Sodium phosphates Tamsulosin	Acyclovir Benzoin-aloe vera-storax-tolu balsam Bupivacaine Chlorothiazide Diatrizoate meglumine-Diatrizoate sodium Dutasteride Ertapenem Fluticasone Gentamicin Glucose Hydrocodone Linezolid Magnesium oxide Metformin Methylprednisolone Nicotine Prasugrel Racemic epinephrine Sotalol Sucralfate Theophylline Valacyclovir

**Table 2:** Cluster 6—medications unassigned through restricted boltzman machine.

Acetaminophen Acetazolamide Acetylcysteine Albumin Albuterol sulfate Allopurinol Alprazolam Alvimopan Amantadine Aminocaproic acid Amlodipine Ammonium lactate Amoxicillin Apixaban Arformoterol Argatroban Aripiprazole Artificial tears Ascorbic acid Atenolol Atropine Azathioprine Azithromycin Aztreonam Bacitracin Baclofen Balanced salt irrigation solution Banana bag Belladonna alkaloids-opium Bendamustine Benzocaine Benzonatate Benztropine Bicalutamide Bisacodyl Brentuximab vedotin Brimonidine Bromocriptine Budesonide Bupropion Butalbital-acetaminophen-caffeine Butamben-tetracaine-benzocaine Calcitonin Calcitriol Calcium acetate Calcium chloride Calcium citrate-vitamin d3	Calcium gluconate Carboplatin Carvedilol Cefepime Ceftaroline Ceftazidime Ceftriaxone Celecoxib Cellulose Cephalexin Chlordiazepoxide Chlorpromazine Chlorthalidone Cholestyramine-aspartame Cilostazol Cinacalcet Ciprofloxacin Cisatracurium Cladribine Clevidipine Clobetasol Clonazepam Clonidine Clopidogrel Colchicine Collagenase clostridium histolyticum Cyanocobalamin Cyclobenzaprine Cyclosporine Cytarabine Dantrolene Daptomycin Desmopressin Dexamethasone Dexmedetomidine Dextromethorphan-guaifenesin Diazepam Dibucaine Diclofenac Digoxin Diltiazem Diphenhydramine Diphenoxylate-atropine Dipyridamole Divalproex Dobutamine Donepezil	Dornase alfa Dorzolamide Doxazosin Doxycycline Dronabinol Duloxetine Econazole Enalapril maleate Enalaprilat Enoxaparin Epinephrine Epoetin alfa Eptifibatide Escitalopram Esmolol Ethacrynate sodium Ethacrynic acid Etomidate Eye preparations Ezetimibe Factor VIIa Famotidine Fat emulsion Fenofibrate Finasteride Flecainide Fluconazole Fludrocortisone Fluorometholone Fluoxetine Folic acid Fondaparinux Formoterol fumarate Fosaprepitant Fosfomycin tromethamine Fosphenytoin Furosemide Gabapentin Gadobenate dimeglumine Gadoterate meglumine Glimepiride Glipizide Glucagon Glycerin Glycopyrrolate Guaifenesin Guar gum oral packet Haloperidol
Heparin Hydralazine Hydrochlorothiazide Hydromorphone Hydroxyzine Ibuprofen Immune globulin (IgG) Insulin Iodixanol Iohexol Iopamidol Ipratropium Iron sucrose Isoproterenol infusion Isosorbide dinitrate Isosorbide mononitrate er Ketamine Ketorolac Labetalol Lacosamide Lactase Lactated ringers Lactulose Lamotrigine Lanthanum Latanoprost Levalbuterol Levetiracetam Levofloxacin Levothyroxine Lidocaine Lipase-protease-amylase Liraglutide Lisinopril Lithium carbonate Loperamide Loratadine Lorazepam Losartan Lovastatin Magnesium citrate oral solution Magnesium hydroxide Magnesium sulfate Mannitol Matrix hemostatic sealant Medroxyprogesterone Meloxicam Memantine Menthol Meperidine Methylnaltrexone	Meropenem Potassium phosphate Pramipexole Prednisolone acetate Prednisolone sodium phosphate Prednisone Pregabalin Prenatal vitamin with calcium no.72-iron Prochlorperazine Promethazine Propofol Propranolol Protamine Prothrombin complex (kcentra) intermittent infusion Pyridostigmine bromide Pyridoxine Quetiapine Raltegravir Ranolazine Rasburicase Remdesivir Rifampin Rifaximin Risperidone Rizatriptan Rocuronium Ropinirole Rosuvastatin Saliva stimulant agents Sertraline Sevelamer Silver nitrate Simethicone Smog enema Sodium bicarbonate Sodium ferric gluconate Sodium hypochlorite Sodium polystyrene sulfonate Spironolactone Succinylcholine chloride Sucralfate Sugammadex Sulfamethoxazole Tacrolimus Methadone Methimazole Methotrexate sodium Methylene blue	Methylphenidate Metoclopramide Metronidazole Micafungin Midazolam Milrinone Minocycline Mometasone-formoterol Morphine Mupirocin Mycophenolate Naloxone Naproxen Nimodipine Nintedanib Nitroglycerin Nitroprusside Norepinephrine Nortriptyline Nxstage fluids Nystatin Octreotide Omeprazole Ondansetron Oseltamivir Oxacillin Oxandrolone Oxycodone Oxymetazoline Paclitaxel Papaverine Paroxetine Pentobarbital Perflutre Phenazopyridine Phenobarbital sodium Phenol Phenylephrine Phenytoin sodium extended Phytonadione Piperacillin-tazobactam Posaconazole Potassium & sodium phosphates Potassium chloride Tamoxifen Tbo-filgrastim Teduglutide Terazosin Tetanus-diphtheria toxoids-td Tezacaftor Thiamine
Thyroid (pork) Tiotropium bromide Tobramycin Tocilizumab Topiramate Torsemide Tramadol Triamterene Valganciclovir Valproic acid Valsartan Vancomycin Vasopressin Vecuronium Verapamil Vitamin a Vitamin b Voriconazole Warfarin Zinc sulfate Ziprasidone Zolpidem		

#### II. Patient clustering

For each patient, the frequency of each medication cluster was counted (see Fig. 1). To obtain a normalized medication cluster distribution for each patient, the frequency table was normalized by the total number of medications taken by each patient. This normalized medication cluster distribution was used as a derived feature for patient clustering.

#### Hierarchical agglomerate clustering

The normalized medication cluster distribution was used to cluster patients using Hierarchical Agglomerative Clustering, which builds a tree to represent data with successor nodes^27^. The optimal number of clusters (n = 5) was identified through the use of the unsupervised pipeline, including visual inspection of the dendrogram (see Fig. 1) and silhouette scores analysis, which was used to identify cluster numbers that provided an equal width among clusters where all clusters are found to have an above average silhouette score (see Appendix Fig. 2). Table 3 describes relevant demographic and outcomes information for each cluster. For implementation, scikit-learn 1.0.2 python library was used to obtain a total of five cluster labels.

**Table 3:** Demographic characteristics by patient cluster.

Cluster	1 (N = 234)	2 (N = 201)	3 (N = 115)	4 (N = 247)	5 (N = 194)
Age (years)	61.5 ± 17.5	61.1 ± 16.8	67.8 ± 14.9	57.0 ± 18.1	62.4 ± 17.8
Sex (female)	109 (46.5)	84 (41.7)	35 (30.4)	119 (48.1)	81 (41.7)
ICU type					
Medical	73 (31.2)	74 (36.8)	32 (27.8)	133 (53.8)	92 (47.4)
Surgical	16 (6.8)	23 (11.4)	3 (2.6)	35 (14.1)	20 (10.3)
Neurosciences	25 (10.6)	17 (8.4)	16 (13.9)	14 (5.6)	21 (10.8)
Burn	34 (14.5)	12 (5.9)	7 (6.0)	11 (4.4)	6 (3.0)
Other	2 (0.8)	8 (3.9)	2 (1.7)	7 (2.8)	3 (1.5)
Admission diagnosis					
Sepsis/infection	8 (3.4)	23 (11.0)	4 (3.4)	42 (17.0)	20 (10.3)
Pulmonary	29 (12.3)	19 (9.4)	5 (4.3)	49 (19.8)	22 (11.4)
Neoplasm	18 (7.6)	9 (4.4)	5 (4.3)	16 (6.4)	14 (7.2)
Gastrointestinal	15 (6.4)	22 (10.9)	8 (6.9)	27 (10.9)	11 (5.7)
Cardiovascular	67 (28.6)	55 (27.3)	43 (37.3)	30 (12.1)	49 (25.3)
Other	14 (5.9)	17 (8.4)	6 (5.2)	14 (5.6)	8 (4.1)
Renal	13 (5.5)	13 (6.4)	5 (4.3)	9 (3.6)	7 (3.6)
Neurology	24 (10.2)	23 (11.4)	27 (23.4)	36 (14.5)	29 (15.0)
Endocrine	8 (3.4)	0 (0.0)	2 (1.7)	5 (2.0)	9 (4.6)
Trauma	38 (16.2)	20 (9.9)	10 (8.7)	19 (7.6)	24 (12.4)
APACHE II at 24 h	13.0 ± 6.4	15.4 ± 6.3	11.3 ± 4.6	16.3 ± 6.6	13.7 ± 5.7
MRC-ICU at 24 h	9.7 ± 7.7	12.3 ± 8.5	5.5 ± 3.8	12.5 ± 7.7	8.7 ± 6.4
Mortality	6 (2.56)	18 (8.96)	3 (2.61)	54 (21.86)	16 (8.25)
Hospital length of stay (days)	8.8 ± 11.9	14.6 ± 20.2	4.8 ± 3.4	15.9 ± 31.1	9.6 ± 9.0
ICU length of stay (days)	4.2 ± 8.9	6.2 ± 8.6	2.4 ± 1.5	7.3 ± 14.3	3.7 ± 3.4
Presence of delirium n (%, total)	41 (18.6, 220)	75 (39.6, 189)	10 (9.4, 106)	115 (53.4, 215)	52 (29.3, 177)
Acute kidney injury n (%, total)	21 (9.1, 232)	39 (19.4)	3 (2.6)	73 (30)	18 (9.3)
Duration of vasopressors support (days)	1.3 ± 0.8	1.8 ± 1.5	1.0 ± 0.0	1.8 ± 1.7	1.3 ± 0.5
Presence of mechanical ventilation	54 (23.0)	89 (44.2)	3 (2.6)	122 (49.3)	44 (22.6)
Duration of mechanical ventilation (days)	1.6 ± 3.1	5.3 ± 9.6	2.7 ± 3.3	8.4 ± 18.1	3.5 ± 4.3
Presence of fluid overload (%, total)	9 (4.5, 199)	26 (13.8, 188)	4 (4.2, 94)	52 (23.9, 217)	14 (8.6, 162)

Data are presented as n (%) or mean ± standard deviation (SD) unless otherwise stated.

##### Validation of clusters

Upon selection of the optimal number of clusters, the validity of these clusters as clinically meaningful subgroups was assessed via surrogate validation conducted by comparing patient outcomes with medication data to see if clinically relevant characteristics were distinguishable.

Wilcoxon rank sum and signed rank tests were performed for continuous characteristics. Fisher's Exact tests were performed for categorical characteristics. Holm’s adjustment of p-values was applied to the comparisons within each outcome to control the familywise error rates. Permutation multivariate analysis of variance (MANOVA) was also used to confirm if the clusters were significantly different considering all clinical outcomes simultaneously^28^. Significance was assessed at p-value < 0.05.

## Results

From the original 1000 patients, a total of 991 patients were included in the analysis with nine excluded due to being repeat ICU admissions. Demographic features are summarized in Table 4 with additional information about the health system provided in Appendix Table 2. The average was 61.2 years old (SD 17.5) with 43% female sex. The patients were managed in the medical ICU 40.7% of the time followed by 9.8% in the surgical and 9.4% in the neurosciences ICU. The mean APACHE II score at 24 h was 14.2 (SD 6.3). The frequency of use for each medication in the analysis is provided in Appendix Table 3, with the top ten medications used including sodium chloride, acetaminophen, potassium chloride, heparin, fentanyl, magnesium sulfate, insulin, furosemide, pantoprazole, and vancomycin.

**Table 4:** Summary of patient population.

Feature	N = 991
Age	61.2 (17.5)
Female	428 (43.2)
ICU type	
Medical	404 (40.7)
Cardiac	305 (30.8)
Surgical	97 (9.8)
Neurosciences	93 (9.4)
Burn	70 (7.1)
Other	22 (2.2)
Admission diagnosis	
Sepsis/infection	97 (9.8)
Pulmonary	124 (12.5)
Neoplasm	62 (6.3)
Gastrointestinal	83 (8.4)
Cardiovascular	244 (24.6)
Dermatology	59 (6.0)
Renal	47 (4.7)
Neurology	139 (14.0)
Endocrine	24 (2.4)
Trauma	111 (11.2)
APACHE II at 24 h	14.2 (6.3)
MRC-ICU at 24 h	10.2 (7.6)
Mortality	97 (9.8)
Hospital length of stay (days)	11.4 (19.7)
ICU length of stay (days)	5.1 (9.5)
Presence of delirium during ICU stay (days)	293 (29.6)
Presence of AKI during ICU stay	151 (25.2)
Duration of vasopressors support (days)	0.5 (1.0)
Presence of mechanical ventilation	318 (32.1)
Duration of mechanical ventilation (days)	5.6 (12.8)
Presence of fluid overload	105 (12.2)

Data are presented as n (%) or mean ± standard deviation (SD) unless otherwise stated.

### Comparison of patient and medication clusters

**Figure 2:**
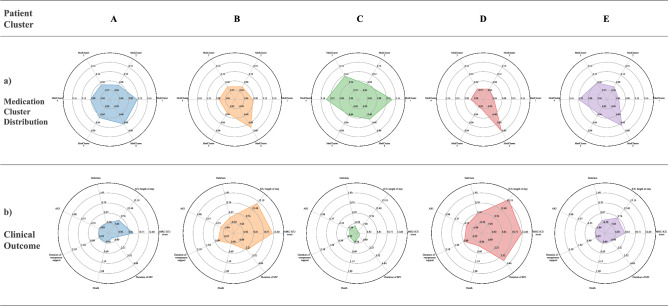
Radial plot distributions in each patient cluster. (**a**) Radial plot of the mean medication cluster distribution in each patient cluster. Patient Cluster 1 has a well-rounded distribution overall when compared to other patient clusters without any outstanding distribution of a particular medication cluster comparably. In contrast, Patient Cluster 4 notably has a high distribution in Medication Cluster 6. (**b**) Radial plot of the mean clinical outcomes in each patient cluster. The lower the mean value, the less severe the outcome was for each clinical outcome category. Thus, Patient Cluster 3 and 5 can be interpreted to have the least serious outcomes while Patient Cluster 2 and 4 generally had worse outcomes.

Five patient clusters were identified through the use of the unsupervised pipeline. Additionally, the silhouette scores analysis plot further suggested a cluster number of 5 provides an equal width between clusters with all clusters having an above average silhouette score (see Appendix Fig. 2). Table 3 describes relevant demographic and outcomes information for each cluster, and Fig. 1 provides a visualization of the distribution of patient clusters by medication clusters and patient outcomes, with lower mean values indicating less severe outcomes. Patient Cluster 1 had a well-rounded distribution overall when compared to other patient clusters and did not have any distinctive distribution for a particular medication cluster. In contrast, Patient Cluster 4 had a high distribution in Medication Cluster 6. Figure 2 summarizes the mean medication cluster distribution for each patient cluster, with the mean medication cluster distribution for each patient cluster provided in Appendix Table 4.

### Comparison of patient clusters by clinical outcomes

**Figure 3:**
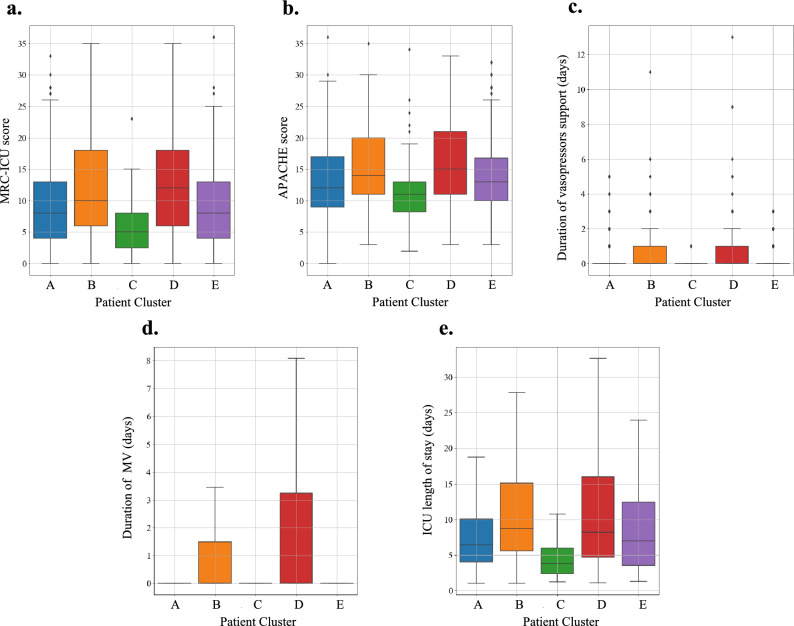
Boxplots of MRC-ICU, APACHE II, and patient outcomes by patient cluster*.* (**a**) MRC-ICU score evaluated at 24 h. (**b**) APACHE score evaluated at 24 h. (**c**) Total days of vasopressor support patient received during admission. (**d**) Total days patient was on mechanical ventilation. (**e**) total days in the ICU. For panel d and e, outliers have been removed to improve visibility of the distribution (full box plots are available in the Appendix).

**Figure 4:**
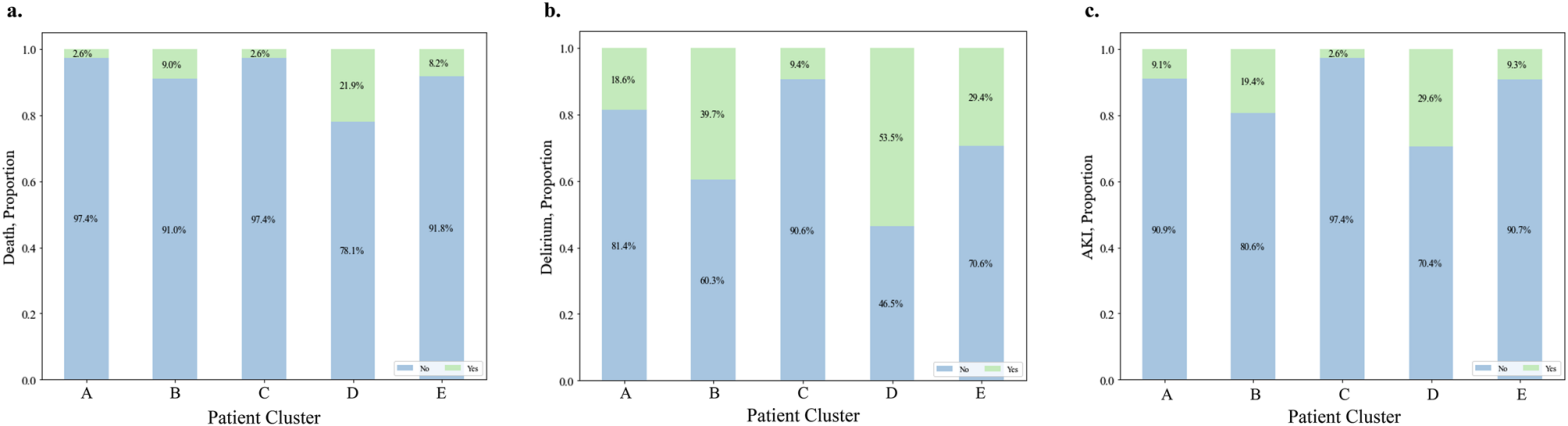
Stacked bar plots showing proportion of patient outcome (categorical) by patient cluster. Any patients with unknown or unreported outcome were removed for analysis.

Patient Cluster 3 and 5 had the least serious outcomes while Patient Cluster 2 and 4 generally had worse patient outcomes. Box plots of outcomes by patient clusters are presented in Fig. 3. For medication clustering purposes, we trained the RBM^25,26^ to learn the high dimensional and non-linear nature among medication assignments based on the co-occurrence of medications for each patient. The default hyperparameters for implementation were used based on the works of Chen^26^. A notable finding was that Patient Clusters 2 and 4 had no statistically significant difference in ICU length of stay, duration of mechanical ventilation, or duration of vasopressor use, but their mortality differed significantly (9.0% vs. 21.9%, p < 0.0018). Patient Cluster 4 had a mortality rate of 21.9% compared with the rest of the clusters ranging between 2.5 and 9% (see Fig. 4). Patient Cluster 4 also had the highest number of outliers (see Appendix Fig. 3). The difference of ICU duration between Patient Clusters 1 and 5 and Patient Clusters 2 and 4 were statistically insignificant. Significance of the differences between patient clusters are summarized in Table 5. Permutation MANOVA further confirmed these differences (p < 0.001) (see Appendix Table 5).

**Table 5:** Pair-wise comparison of differences in patient outcomes by patient cluster.

1st Cluster	2nd Cluster	Continuous outcomes					Categorical outcomes			
		Length of stay (days)	Duration of mechanical ventilation (days)	Duration of vasopressor support (days)	APACHE II score (first 24 h)	MRC-ICU	Death	Acute kidney injury	Delirium	Mechanical ventilation
1	2	< 0.0001	< 0.0001	0.0002	0.0003	0.0012	0.0312	0.0130	< 0.0001	< 0.0001
1	3	< 0.0001	< 0.0001	0.0370	0.1695	< 0.0001	1.0000	0.0760	0.0692	< 0.0001
1	4	0.0009	< 0.0001	< 0.0001	< 0.0001	< 0.0001	< 0.0001	< 0.0001	< 0.0001	< 0.0001
1	5	0.4803	0.6110	0.8065	0.1904	1.0000	0.0689	1.0000	0.0380	0.6700
2	3	< 0.0001	< 0.0001	< 0.0001	< 0.0001	< 0.0001	0.1353	< 0.0001	< 0.0001	< 0.0001
2	4	0.4803	0.0570	0.3478	0.1904	1.0000	0.0018	0.0630	0.0278	0.6700
2	5	0.0007	< 0.0001	0.0002	0.0328	0.0002	1.0000	0.0210	0.0692	< 0.0001
3	4	< 0.0001	< 0.0001	< 0.0001	< 0.0001	< 0.0001	< 0.0001	< 0.0001	< 0.0001	< 0.0001
3	5	< 0.0001	< 0.0001	0.0583	0.0015	< 0.0001	0.1536	0.0760	0.0003	< 0.0001
4	5	0.0245	< 0.0001	< 0.0001	0.0002	< 0.0001	0.0009	< 0.0001	< 0.0001	< 0.0001

Wilcoxon rank sum and signed rank tests were performed for continuous variables. Fisher's Exact tests were performed for categorical outcomes. Holm’s adjustment of p-values was applied to the comparisons within each outcome to control the familywise error rates.

## Discussion

In the first unsupervised machine learning analysis of critically ill patients and their medication regimens, five unique patient clusters were identified with significant differences in severity of illness and outcomes. Six pharmacophenotypes were identified, and each patient cluster displayed a unique distribution of these six pharmacophenotypes. This study is the first to apply AI to the complete medication list of ICU patients and demonstrates the ability to appropriately categorize patients with their outcomes, which lays the groundwork for future investigations.

Unsupervised machine learning methods have been previously explored for the derivation of distinct clinical phenotypes and biological endotypes^29–31^. Prior approaches have frequently used methods such as Latent Class Analysis (LCA) to identify clusters that are separatable by the input data. Latent Class Analysis is a set of Finite Mixture Models, which utilize a probablistic model-based clustering approach, in which each cluster are characterized on a probabilistic distribution rather than their centroid-based distance (such as with k-Means). Thus, each cluster has a probability of association, rather than a clear membership assignment. Due to the probabilistic nature of the class assignment, it may be difficult to derive instance-level associations, thus a single instance may belong marginally to multiple classes^32,33^. Alternatively, k-means allows for a characterization of clusters driven by centroid-based distances, allowing for a quantitative estimate of the membership^34^. Due to the heterogeneity of the input data, our goal in this work was to distinguish between a finite set of classes and better understand their distance-based profile when medications are utilized in the derivation rather than a probabilistic model of their likelihood.

**Figure 5:**
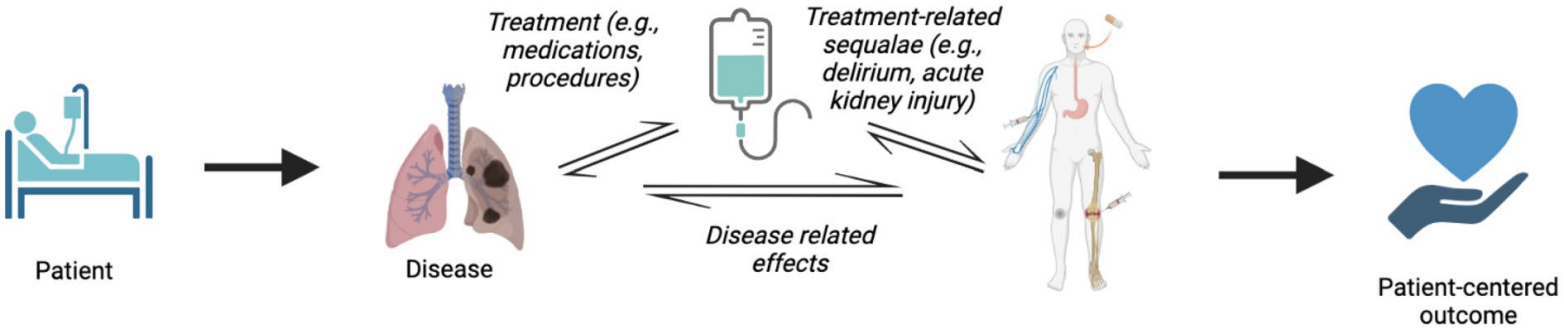
Patient–Treatment–Outcome Pathway. The unique interactions of medication interventions with patient disease must be accounted for when predicting or studying patient-centered outcomes.

Critically ill patients are medically complex with requisitely complex medication regimens. The significant challenges to characterizing complex, heterogeneous ICU medications in a meaningful way to drive clinical decision making parallel the challenges of managing and researching complex ICU syndromes like ARDS and sepsis. Indeed, it was reported that 62 of 76 randomized-controlled trials evaluating mortality showed no significant difference and just three of those positive studies have been accepted into practice^35^. Similar findings have been paralleled in ARDS^36^. Thoughtful editorials on this statistically unlikely preponderance of negative results have been published, and although common reasons for negative ICU studies likely account for some of these negative trials (e.g., underpowered studies, need for the use of a more conservative p-value cut-off), these statistical explanations ignore the potentially biological ones, wherein the target of an intervention is absent due to limitations in specificity of diagnosis, animal models of disease, or understanding of underlying pathophysiology^37–39^. Additionally, we would like to propose another relevant driver of patient outcomes that is generally unaccounted for in both RCTs and predictive modeling studies: the complete ICU medication regimen. Traditionally, ICU medications are often thought to be direct results of critical illness (e.g., a septic patient with a high lactate is prescribed broad-spectrum antibiotics and vasopressors). However, this simplified pathway does not incorporate that ICU medications are also independent risk factors for ICU complications that worsen patient outcomes (e.g., this septic patient develops acute kidney injury, which may be due to the shock state or the use of nephrotoxic medications or the combination of disease plus medication). Thus, when making medication-related decisions, medications must be thought of as both treatments and causes of outcomes (see Fig. 5). Aside from overt medication errors, ICU medications are also associated with significant ICU complications that increase risk of mortality and length of stay including ICU delirium, fluid overload, acute kidney injury, etc^40–43^. Ultimately, the benefits to medications used to manage critical illness must be balanced by mitigating the harms of those same treatments. Because medications in the ICU are always used in combination with other medications and interventions, identifying which medication and which medication combinations confer less risk for ICU complications has the potential to guide safer medication use. However, the dynamic relationships among patients, medications, and outcomes have been difficult to delineate given the inherent complexities and largess of ICU patient care and the data generated in that process. Given that medications are independent risk factors, a future direction for this type of clustering analysis is to generate a dataset capable of matching patients by demographic and clinical presentation variables and then compare outcomes of those with similar vs. different pharmacophenotypes. Moreover, this type of analysis will be aided by a multi-center design that improves external generalizability given that medication regimens may reflect local practices or other healthcare team related origins, instead of purely patient pathophysiology.

Phenotyping, especially when conducted through artificial intelligence methods, has significant potential to overcome challenges related to heterogeneity and non-linear relationships present in critically ill populations. When Calfee et al. used biomarker-based phenotyping in a re-analysis of a large randomized-controlled trial evaluating simvastatin (a trial that notably had previously shown negative results), significantly different treatment response wherein one phenotype showed mortality benefit from simvastatin was observed^44^. Moreover, these ARDS phenotypes also showed differential treatment response to fluid management strategy^45^. Similarly, AI methods have demonstrated the presence of unique clusters in shock, sepsis, and fluid overload^46,47^. Notably, Seymour et al. demonstrated that the results of three major randomized controlled trials were sensitive to the sepsis phenotypes they derived via unsupervised machine learning methods. Another series of shock sub-phenotypes was characterized by features associated with common ICU interventions (e.g., “well resuscitated” or “still hypovolemic”) that upon appropriate validation could yield highly relevant insights for bedside decision-making^47^. Our cluster pipeline driven by unsupervised feature learning using RBM and hierarchical clustering categorized medications into five unique clusters, with the remaining medications creating a sixth category. Of the patient clusters, Clusters 2 and 4 had the highest acuity, as measured by APACHE II. This high acuity was accompanied by significantly worse outcomes, including length of stay, ICU length of stay, presence of delirium and fluid overload, and need for mechanical ventilation. Interestingly, despite being similar, Patient Cluster 4 had a mortality rate over twice as high as Cluster 2. When evaluating the distribution of pharmacophenotypes, Cluster 4 had the highest density of Medication Cluster 6 and limited representation among the other five clusters. This particular pharmacophenotype contains many of the medications classically associated with ICU care including vasopressors and broad-spectrum antibiotics. Conversely, Cluster 3 had the lowest severity of illness and best outcomes and also had the lowest density of all the pharmacophenotypes. This suggests a possibility of non-linear relationships between medication regimen complexity and outcomes seen in other analyses^48^. Medication regimen complexity, as measured by the MRC-ICU, has been previously incorporated into ML prediction models along with other relevant patient characteristics and resulted in improved mortality prediction in a small cohort of patients^49^. In this study, medication regimen complexity was highest in Patient Clusters 2 and 4, which is in line with previous investigations of MRC-ICU that used traditional inferential statistics to demonstrate a relationship between increasing medication regimen complexity and increased mortality, length of stay, and fluid overload as well as increased need for critical care pharmacist interventions to optimize the medication regimens^50–55^. Taken together, the methodologies in this study appear to be able to appropriately group degree of critical illness (i.e., severity) with degree of intervention intensity (e.g., mechanical ventilation, medications) with patient outcomes (e.g., mortality). This congruence sets the foundation for future investigations to predict ICU complications based on unique medication combinations that deleteriously affect patient outcomes.

Overall, this evaluation was a proof-of-concept investigation to explore how unsupervised clustering methods may be applied to ICU medications, and while it has novel implications, future evaluations to address certain limitations are warranted that include comparative approaches, larger datasets, and more granular medication information. Comparative evaluations may include matrix factorization or other robust forms of RBM (e.g., Gaussian-Bernoulli RBM)^23^. Only generic drug name was used to describe the medications with dose, route, and other formulation information excluded. Establishing uniform means of describing and comparing ICU medication dosing strategies (e.g., a common data model) and validating these approaches in external datasets remains an area of future work. We assumed homogeneity across medication regimens; however, in practice this may be a highly complex and noisy interaction: therefore, in future work, we seek to utilize Trust Discover platforms to generalize pharmacotherapy profiles that are normalized independent of clinician and institutional bias^56^. Causal inference cannot be assessed by the current study, so it is unknown whether the high mortality observed in Patient Cluster 4 was partly caused by the unique distribution of pharmacophenotypes versus other factors (although notably, Cluster 4 shared similarities among groups). Even with these limitations, this analysis marks the first time the complete medication profile has been incorporated into outcomes analysis for ICU patients. Future analyses with more granular pharmacophenotype groupings or more programmed directives incorporating data from a myriad of ICUs and centers may improve the face validity form the viewpoint of the clinician for these pharmacophenotypes.

## Conclusion

The medication regimens of critically ill patients have unique pharmacophenotypes. Given the significant role of medication therapy in patient outcomes, delineating the complex relationships among patients, medications, and outcomes using artificial intelligence warrants future investigation.

### Supplementary Information

Supplementary Information.

## Data Availability

Data will be made available upon request by the editor and/or reviewers from the corresponding author.
