# Supplementary material for: Cluster analysis driven by unsupervised latent feature learning of medications to identify novel pharmacophenotypes of critically ill patients
Source: Sci Rep. 2023 Sep 20;13:15562. doi: 10.1038/s41598-023-42657-2 (PMC10511715; doi:10.1038/s41598-023-42657-2)

**Supplementary Table 1.** Outcome labels

| **Feature Name** | **Description** | **Categorical Label** | **Numeric Label** |
| --- | --- | --- | --- |
| **Death** | Patient death at discharge | Alive | 0 |
|  |  | Death | 1 |
| **Delirium** | Development of Delirium during ICU admission | Unknown | -1 |
|  |  | Negative | 0 |
|  |  | Positive | 1 |
| **Acute kidney injury** | Development of acute kidney injury during ICU admission | No | 0 |
|  |  | Yes | 1 |
| **ICU length of stay** | Total number of days in the intensive care unit (ICU) | Continuous numeric value | |
| **Duration of mechanical ventilation** | Duration of patient being on mechanical ventilation for respiratory support at ICU | Continuous numeric value | |
| **First mechanical ventilation** | Categorization of the first mechanical ventilation onset | None | -1 |
|  |  | Before 24 hours | 0 |
|  |  | After 24 hours | 1 |
| **Duration of vasopressors support** | Duration of patient administered vasopressors | Continuous numeric value | |

**Supplementary Table 2.** Hospitals represented in the patient population studied.

| Hospital | N = 991 |
| --- | --- |
| Pardee Hospital | 1 |
| UNC Hospitals Hillsborough | 26 |
| UNC REX | 388 |
| UNC Health Wayne | 16 |
| UNC Medical Center | 560 |

**Supplementary Table 3.** Medications Frequency

| **Medications (Alphabetical Order)** | **Frequency n, (%)** | **Medication (Descending order by frequency percentage)** | **%** |
| --- | --- | --- | --- |
| ACETAMINOPHEN | 722 (72.9%) | SODIUM CHLORIDE | 79.7 |
| ACETAZOLAMIDE | 30 (3%) | ACETAMINOPHEN | 72.9 |
| ACETYLCYSTEINE | 9 (0.9%) | POTASSIUM CHLORIDE | 64.5 |
| ACYCLOVIR | 15 (1.5%) | HEPARIN | 64.3 |
| ADENOSINE | 5 (0.5%) | FENTANYL | 59.9 |
| ALBUMIN | 230 (23.2%) | MAGNESIUM SULFATE | 52.2 |
| ALBUTEROL SULFATE | 243 (24.5%) | INSULIN | 47.7 |
| ALLOPURINOL | 39 (3.9%) | FUROSEMIDE | 47.1 |
| ALPRAZOLAM | 30 (3%) | PANTOPRAZOLE | 46.2 |
| ALTEPLASE | 77 (7.8%) | VANCOMYCIN | 45.9 |
| ALUMINUM-MAG HYDROXIDE-SIMETHICONE | 50 (5%) | ASPIRIN | 44.7 |
| ALVIMOPAN | 0 (0%) | ONDANSETRON | 42.6 |
| AMANTADINE HCL | 4 (0.4%) | LACTATED RINGERS | 42.1 |
| AMINOCAPROIC ACID | 17 (1.7%) | POLYETHYLENE GLYCOL | 41.2 |
| AMIODARONE | 93 (9.4%) | IOHEXOL | 40.7 |
| AMITRIPTYLINE | 7 (0.7%) | OXYCODONE | 39.7 |
| AMLODIPINE | 164 (16.5%) | LIDOCAINE | 39.3 |
| AMMONIUM LACTATE | 5 (0.5%) | DOCUSATE SODIUM | 39.1 |
| AMOXICILLIN | 54 (5.4%) | ENOXAPARIN | 38.8 |
| AMPHOTERICIN B LIPOSOMAL (AMBISOME) IVPB IN | 1 (0.1%) | MORPHINE | 38.5 |
| AMPICILLIN | 64 (6.5%) | MIDAZOLAM | 38.3 |
| ANAKINRA | 1 (0.1%) | METOLAZONE | 37.4 |
| APIXABAN | 79 (8%) | MAGNESIUM OXIDE | 36.5 |
| ARFORMOTEROL | 34 (3.4%) | ATORVASTATIN | 34.8 |
| ARGATROBAN | 1 (0.1%) | MOUTHWASH | 33.5 |
| ARIPIPRAZOLE | 8 (0.8%) | DEXTROSE | 31.1 |
| ARTIFICIAL TEARS | 51 (5.1%) | FAMOTIDINE | 30.8 |
| ASCORBIC ACID (VITAMIN C) | 93 (9.4%) | LORAZEPAM | 30.6 |
| ASPIRIN | 443 (44.7%) | PROPOFOL | 28.7 |
| ATENOLOL | 14 (1.4%) | IPRATROPIUM | 27 |
| ATORVASTATIN | 345 (34.8%) | HYDRALAZINE | 26.1 |
| ATOVAQUONE | 4 (0.4%) | CEFEPIME | 25.2 |
| ATROPINE | 35 (3.5%) | ALBUTEROL SULFATE | 24.5 |
| AZATHIOPRINE | 6 (0.6%) | HYDROMORPHONE | 24.2 |
| AZELASTINE | 3 (0.3%) | SENNOSIDES | 23.7 |
| AZITHROMYCIN | 80 (8.1%) | ALBUMIN | 23.2 |
| AZTREONAM | 21 (2.1%) | BACITRACIN | 20.9 |
| BACITRACIN | 207 (20.9%) | CALCIUM GLUCONATE | 20.9 |
| BACLOFEN | 18 (1.8%) | MULTIVITAMIN | 20.8 |
| BALANCED SALT IRRIGATION SOLUTION (BSS PLAIN) | 10 (1%) | MELATONIN | 20.5 |
| BANANA BAG | 2 (0.2%) | NOREPINEPHRINE | 20.1 |
| BARIUM SULFATE | 18 (1.8%) | CEFAZOLIN | 19.4 |
| BASILIXIMAB INFUSION | 6 (0.6%) | LISINOPRIL | 19.3 |
| BELLADONNA ALKALOIDS-OPIUM | 3 (0.3%) | GABAPENTIN | 19.1 |
| BENDAMUSTINE (BENDEKA) IVPB | 1 (0.1%) | PIPERACILLIN-TAZOBACTAM | 18.8 |
| BENZOCAINE | 2 (0.2%) | CEFTRIAXONE | 17.4 |
| BENZOIN-ALOE VERA-STORAX-TOLU BALSAM | 1 (0.1%) | LABETALOL | 16.8 |
| BENZONATATE | 14 (1.4%) | AMLODIPINE | 16.5 |
| BENZTROPINE | 1 (0.1%) | DEXMEDETOMIDINE | 16.5 |
| BICALUTAMIDE | 2 (0.2%) | DIPHENHYDRAMINE | 16.5 |
| BIOTIN | 1 (0.1%) | SODIUM BICARBONATE | 15.4 |
| BISACODYL | 149 (15%) | CARVEDILOL | 15.3 |
| BIVALIRUDIN | 2 (0.2%) | TRAMADOL | 15.1 |
| BRENTUXIMAB VEDOTIN (ADCETRIS) IVPB | 2 (0.2%) | BISACODYL | 15 |
| BRIMONIDINE | 9 (0.9%) | LEVOFLOXACIN | 14.3 |
| BROMOCRIPTINE | 3 (0.3%) | METRONIDAZOLE | 14.3 |
| BUDESONIDE | 74 (7.5%) | THIAMINE HCL | 14.1 |
| BUMETANIDE | 73 (7.4%) | PHENYLEPHRINE | 13.1 |
| BUPIVACAINE (PF) | 61 (6.2%) | CLOPIDOGREL | 12.2 |
| BUPRENORPHINE | 4 (0.4%) | NITROGLYCERIN | 12.1 |
| BUPROPION HCL | 14 (1.4%) | VASOPRESSIN | 11.8 |
| BUSPIRONE | 19 (1.9%) | EPINEPHRINE | 11.5 |
| BUTALBITAL-ACETAMINOPHEN-CAFFEINE | 27 (2.7%) | METHYLPREDNISOLONE | 11.5 |
| BUTAMBEN-TETRACAINE-BENZOCAINE | 1 (0.1%) | PREDNISONE | 11.3 |
| CALCITONIN (SALMON) | 4 (0.4%) | PROMETHAZINE | 11.2 |
| CALCITRIOL | 10 (1%) | IODIXANOL | 11 |
| CALCIUM ACETATE | 12 (1.2%) | FOLIC ACID | 10.6 |
| CALCIUM CARBONATE | 95 (9.6%) | LEVOTHYROXINE | 10.4 |
| CALCIUM CHLORIDE | 48 (4.8%) | CALCIUM CARBONATE | 9.6 |
| CALCIUM CITRATE-VITAMIN D3 | 2 (0.2%) | LEVETIRACETAM | 9.5 |
| CALCIUM GLUCONATE | 207 (20.9%) | THROMBIN | 9.5 |
| CARBIDOPA | 8 (0.8%) | AMIODARONE | 9.4 |
| CARBOPLATIN (PARAPLATIN) IVPB (BY AUC) | 3 (0.3%) | ASCORBIC ACID (VITAMIN C) | 9.4 |
| CARTEOLOL | 1 (0.1%) | KETOROLAC | 9.4 |
| CARVEDILOL | 152 (15.3%) | METOCLOPRAMIDE | 9.4 |
| CEFAZOLIN | 192 (19.4%) | POTASSIUM PHOSPHATE IVPB | 9.3 |
| CEFDINIR | 18 (1.8%) | TAMSULOSIN | 9.2 |
| CEFEPIME | 250 (25.2%) | FLUTICASONE | 8.9 |
| CEFTAROLINE IVPB IN | 4 (0.4%) | MUPIROCIN | 8.9 |
| CEFTAZIDIME | 11 (1.1%) | NYSTATIN | 8.9 |
| CEFTRIAXONE | 172 (17.4%) | HYDROCORTISONE | 8.5 |
| CEFUROXIME (ZINACEF) | 11 (1.1%) | HALOPERIDOL | 8.4 |
| CELECOXIB | 24 (2.4%) | MEROPENEM | 8.3 |
| CELLULOSE, OXIDIZED REG | 53 (5.3%) | DOPAMINE | 8.2 |
| CEPHALEXIN | 31 (3.1%) | POTASSIUM, SODIUM PHOSPHATES | 8.2 |
| CETIRIZINE | 20 (2%) | AZITHROMYCIN | 8.1 |
| CHLORDIAZEPOXIDE | 10 (1%) | APIXABAN | 8 |
| CHLOROTHIAZIDE SODIUM | 25 (2.5%) | DEXAMETHASONE | 7.9 |
| CHLORPROMAZINE | 2 (0.2%) | ALTEPLASE | 7.8 |
| CHLORTHALIDONE | 11 (1.1%) | GUAIFENESIN | 7.8 |
| CHOLECALCIFEROL (VITAMIN D3) | 69 (7%) | DILTIAZEM | 7.7 |
| CHOLESTYRAMINE-ASPARTAME | 1 (0.1%) | BUDESONIDE | 7.5 |
| CILOSTAZOL | 2 (0.2%) | BUMETANIDE | 7.4 |
| CINACALCET | 6 (0.6%) | DOXYCYCLINE | 7.4 |
| CIPROFLOXACIN | 16 (1.6%) | NICARDIPINE | 7.4 |
| CISATRACURIUM | 12 (1.2%) | PRAVASTATIN | 7.3 |
| CITALOPRAM | 33 (3.3%) | TRAZODONE | 7.3 |
| CITRATE DEXTROSE SOLUTION | 4 (0.4%) | GADOBENATE DIMEGLUMINE | 7.2 |
| CLADRIBINE (LEUSTATIN) IVPB | 1 (0.1%) | ETOMIDATE | 7.1 |
| CLEVIDIPINE | 8 (0.8%) | LOSARTAN | 7.1 |
| CLINDAMYCIN | 44 (4.4%) | CHOLECALCIFEROL (VITAMIN D3) | 7 |
| CLOBAZAM | 1 (0.1%) | OXYCODONE-ACETAMINOPHEN | 6.9 |
| CLOBETASOL | 3 (0.3%) | HYDROXYZINE HCL | 6.7 |
| CLONAZEPAM | 37 (3.7%) | IBUPROFEN | 6.6 |
| CLONIDINE | 34 (3.4%) | AMPICILLIN | 6.5 |
| CLOPIDOGREL | 121 (12.2%) | VERAPAMIL | 6.5 |
| CODEINE | 10 (1%) | PERFLUTRE | 6.3 |
| COLCHICINE | 21 (2.1%) | BUPIVACAINE (PF) | 6.2 |
| COLLAGENASE CLOSTRIDIUM HISTOLYTICUM | 5 (0.5%) | PHYTONADIONE (VITAMIN K1) | 6.1 |
| CONJUGATED ESTROGENS | 2 (0.2%) | OMEPRAZOLE | 5.9 |
| CYANOCOBALAMIN (VIT B-12) | 25 (2.5%) | GELATIN | 5.8 |
| CYCLOBENZAPRINE | 27 (2.7%) | LACTULOSE | 5.8 |
| CYCLOSPORINE | 3 (0.3%) | OLANZAPINE | 5.7 |
| CYCLOSPORINE MODIFIED | 1 (0.1%) | FERROUS SULFATE | 5.5 |
| CYTARABINE (ARA-C) IVPB | 1 (0.1%) | HYDROCODONE | 5.5 |
| DANTROLENE | 1 (0.1%) | SILVER SULFADIAZINE | 5.5 |
| DAPTOMYCIN (CUBICIN) IVPB IN | 13 (1.3%) | AMOXICILLIN | 5.4 |
| DAUNORUBICIN (CERUBIDINE) SYRINGE | 1 (0.1%) | FLUCONAZOLE | 5.4 |
| DESMOPRESSIN (DDAVP) | 17 (1.7%) | CELLULOSE, OXIDIZED REG | 5.3 |
| DEXAMETHASONE | 78 (7.9%) | GENTAMICIN | 5.2 |
| DEXMEDETOMIDINE | 164 (16.5%) | ARTIFICIAL TEARS | 5.1 |
| DEXTROMETHORPHAN-GUAIFENESIN | 25 (2.5%) | LOPERAMIDE | 5.1 |
| DEXTROSE | 308 (31.1%) | SODIUM PHOSPHATES | 5.1 |
| DIATRIZOATE MEGLUMINE-DIATRIZOATE SODIUM | 1 (0.1%) | ALUMINUM-MAG HYDROXIDE-SIMETHICONE | 5 |
| DIAZEPAM | 44 (4.4%) | EYE PREPARATIONS | 5 |
| DIBUCAINE | 2 (0.2%) | QUETIAPINE | 5 |
| DICLOFENAC | 24 (2.4%) | METFORMIN | 4.9 |
| DIGOXIN | 34 (3.4%) | CALCIUM CHLORIDE | 4.8 |
| DILTIAZEM | 76 (7.7%) | PREGABALIN | 4.8 |
| DIPHENHYDRAMINE | 164 (16.5%) | DOBUTAMINE | 4.7 |
| DIPHENOXYLATE-ATROPINE | 8 (0.8%) | SPIRONOLACTONE | 4.7 |
| DIPYRIDAMOLE | 1 (0.1%) | ZINC SULFATE | 4.5 |
| DIVALPROEX | 10 (1%) | CLINDAMYCIN | 4.4 |
| DOBUTAMINE | 47 (4.7%) | DIAZEPAM | 4.4 |
| DOCUSATE SODIUM | 387 (39.1%) | LORATADINE | 4.1 |
| DONEPEZIL | 11 (1.1%) | MATRIX HEMOSTATIC SEALANT | 4.1 |
| DONOR LEVOTHYROXINE | 1 (0.1%) | SERTRALINE | 4.1 |
| DOPAMINE | 81 (8.2%) | HYDROCHLOROTHIAZIDE | 4 |
| DORNASE ALFA | 7 (0.7%) | MAGNESIUM CITRATE ORAL SOLUTION | 4 |
| DORZOLAMIDE | 13 (1.3%) | ALLOPURINOL | 3.9 |
| DOXAZOSIN | 4 (0.4%) | WARFARIN | 3.9 |
| DOXYCYCLINE | 73 (7.4%) | TIOTROPIUM BROMIDE | 3.8 |
| DRONABINOL | 8 (0.8%) | CLONAZEPAM | 3.7 |
| DROXIDOPA | 1 (0.1%) | LINEZOLID | 3.7 |
| DULOXETINE | 36 (3.6%) | PAPAVERINE | 3.7 |
| DUTASTERIDE | 1 (0.1%) | DULOXETINE | 3.6 |
| ECONAZOLE | 2 (0.2%) | NXSTAGE RFP | 3.6 |
| EMOLLIENT COMBO NUMBER | 1 (0.1%) | SIMETHICONE | 3.6 |
| EMTRICITABINE-TENOFOVIR | 1 (0.1%) | ATROPINE | 3.5 |
| ENALAPRIL MALEATE | 3 (0.3%) | PHENOL | 3.5 |
| ENALAPRILAT | 16 (1.6%) | ARFORMOTEROL | 3.4 |
| ENOXAPARIN | 385 (38.8%) | CLONIDINE | 3.4 |
| EPINEPHRINE | 114 (11.5%) | DIGOXIN | 3.4 |
| EPOETIN ALFA | 29 (2.9%) | MIRTAZAPINE | 3.4 |
| EPTIFIBATIDE | 14 (1.4%) | VECURONIUM | 3.4 |
| ERTAPENEM (INVANZ) | 6 (0.6%) | CITALOPRAM | 3.3 |
| ESCITALOPRAM | 29 (2.9%) | MAGNESIUM HYDROXIDE | 3.2 |
| ESMOLOL | 5 (0.5%) | TICAGRELOR | 3.2 |
| ESOMEPRAZOLE MAGNESIUM DR | 9 (0.9%) | CEPHALEXIN | 3.1 |
| ESTRADIOL | 2 (0.2%) | MONTELUKAST | 3.1 |
| ETHACRYNATE SODIUM | 1 (0.1%) | ACETAZOLAMIDE | 3 |
| ETHACRYNIC ACID | 1 (0.1%) | ALPRAZOLAM | 3 |
| ETOMIDATE | 70 (7.1%) | ISOSORBIDE MONONITRATE ER | 3 |
| EXPAREL ADMINISTERED WITHIN | 3 (0.3%) | SUCRALFATE | 3 |
| EYE PREPARATIONS | 50 (5%) | EPOETIN ALFA | 2.9 |
| EZETIMIBE | 11 (1.1%) | ESCITALOPRAM | 2.9 |
| FACTOR VIIA RECOMB (NOVOSEVEN) INTERMITTENT INFUSION | 2 (0.2%) | SULFAMETHOXAZOLE | 2.9 |
| FAMOTIDINE | 305 (30.8%) | FLUOXETINE | 2.8 |
| FAT EMULSION | 8 (0.8%) | GLYCOPYRROLATE | 2.8 |
| FENOFIBRATE | 7 (0.7%) | MENTHOL | 2.8 |
| FENTANYL | 594 (59.9%) | PEG | 2.8 |
| FERROUS SULFATE | 55 (5.5%) | SUCCINYLCHOLINE CHLORIDE | 2.8 |
| FINASTERIDE | 21 (2.1%) | BUTALBITAL-ACETAMINOPHEN-CAFFEINE | 2.7 |
| FLECAINIDE | 4 (0.4%) | CYCLOBENZAPRINE | 2.7 |
| FLUCONAZOLE | 54 (5.4%) | PROPRANOLOL | 2.7 |
| FLUDROCORTISONE | 6 (0.6%) | ROCURONIUM | 2.7 |
| FLUOROMETHOLONE | 3 (0.3%) | SEVELAMER | 2.6 |
| FLUOXETINE | 28 (2.8%) | CHLOROTHIAZIDE SODIUM | 2.5 |
| FLUTICASONE | 88 (8.9%) | CYANOCOBALAMIN (VIT B-12) | 2.5 |
| FOLIC ACID | 105 (10.6%) | DEXTROMETHORPHAN-GUAIFENESIN | 2.5 |
| FONDAPARINUX | 1 (0.1%) | GLYCERIN | 2.5 |
| FORMOTEROL FUMARATE | 3 (0.3%) | MICAFUNGIN IVPB | 2.5 |
| FOSAPREPITANT (EMEND) IVPB | 2 (0.2%) | NEOMYCIN | 2.5 |
| FOSFOMYCIN TROMETHAMINE | 8 (0.8%) | VALACYCLOVIR | 2.5 |
| FOSPHENYTOIN IV | 5 (0.5%) | CELECOXIB | 2.4 |
| FUROSEMIDE | 467 (47.1%) | DICLOFENAC | 2.4 |
| GABAPENTIN | 189 (19.1%) | GADOTERATE MEGLUMINE | 2.4 |
| GADOBENATE DIMEGLUMINE | 71 (7.2%) | ISOSORBIDE DINITRATE | 2.3 |
| GADOTERATE MEGLUMINE | 24 (2.4%) | LATANOPROST | 2.3 |
| GANCICLOVIR (CYTOVENE) IVPB | 6 (0.6%) | LEVALBUTEROL | 2.3 |
| GELATIN | 57 (5.8%) | NALOXONE | 2.2 |
| GENTAMICIN | 52 (5.2%) | ZOLPIDEM | 2.2 |
| GLIMEPIRIDE | 4 (0.4%) | AZTREONAM | 2.1 |
| GLIPIZIDE | 10 (1%) | COLCHICINE | 2.1 |
| GLUCAGON | 7 (0.7%) | FINASTERIDE | 2.1 |
| GLUCOSE | 1 (0.1%) | MIDODRINE | 2.1 |
| GLYCERIN | 25 (2.5%) | MYCOPHENOLATE | 2.1 |
| GLYCOPYRROLATE | 28 (2.8%) | PRASUGREL | 2.1 |
| GUAIFENESIN | 77 (7.8%) | RIVAROXABAN | 2.1 |
| GUAR GUM ORAL PACKET | 18 (1.8%) | CETIRIZINE | 2 |
| HALOPERIDOL | 83 (8.4%) | OXYMETAZOLINE | 2 |
| HEPARIN | 637 (64.3%) | SODIUM POLYSTYRENE SULFONATE | 2 |
| HYDRALAZINE | 259 (26.1%) | BUSPIRONE | 1.9 |
| HYDROCHLOROTHIAZIDE | 40 (4%) | KETAMINE | 1.9 |
| HYDROCODONE | 55 (5.5%) | MANNITOL | 1.9 |
| HYDROCORTISONE | 84 (8.5%) | BACLOFEN | 1.8 |
| HYDROMORPHONE | 240 (24.2%) | BARIUM SULFATE | 1.8 |
| HYDROXYCHLOROQUINE | 11 (1.1%) | CEFDINIR | 1.8 |
| HYDROXYUREA | 1 (0.1%) | GUAR GUM ORAL PACKET | 1.8 |
| HYDROXYZINE HCL | 66 (6.7%) | OCTREOTIDE | 1.8 |
| IBUPROFEN | 65 (6.6%) | SMOG ENEMA | 1.8 |
| IMMUNE GLOB,GAMM(IGG) | 8 (0.8%) | AMINOCAPROIC ACID | 1.7 |
| INDOMETHACIN | 1 (0.1%) | DESMOPRESSIN (DDAVP) | 1.7 |
| INSULIN | 473 (47.7%) | MEPERIDINE (DEMEROL) | 1.7 |
| IODIXANOL | 109 (11%) | PROCHLORPERAZINE MALEATE | 1.7 |
| IOHEXOL | 403 (40.7%) | RIFAXIMIN | 1.7 |
| IOPAMIDOL | 2 (0.2%) | VENLAFAXINE | 1.7 |
| IPRATROPIUM | 268 (27%) | CIPROFLOXACIN | 1.6 |
| IRON SUCROSE | 6 (0.6%) | ENALAPRILAT | 1.6 |
| ISOPROTERENOL INFUSION | 4 (0.4%) | OXYBUTYNIN CHLORIDE | 1.6 |
| ISOSORBIDE DINITRATE | 23 (2.3%) | TACROLIMUS | 1.6 |
| ISOSORBIDE MONONITRATE ER | 30 (3%) | TRIAMCINOLONE | 1.6 |
| KETAMINE | 19 (1.9%) | ACYCLOVIR | 1.5 |
| KETOROLAC | 93 (9.4%) | OSELTAMIVIR | 1.5 |
| LABETALOL | 166 (16.8%) | TORSEMIDE | 1.5 |
| LACOSAMIDE | 13 (1.3%) | TUBERCULIN PPD | 1.5 |
| LACTASE | 1 (0.1%) | ATENOLOL | 1.4 |
| LACTATED RINGERS | 417 (42.1%) | BENZONATATE | 1.4 |
| LACTOBACILLUS | 14 (1.4%) | BUPROPION HCL | 1.4 |
| LACTULOSE | 57 (5.8%) | EPTIFIBATIDE | 1.4 |
| LAMOTRIGINE | 3 (0.3%) | LACTOBACILLUS | 1.4 |
| LANTHANUM | 1 (0.1%) | NIFEDIPINE ER | 1.4 |
| LATANOPROST | 23 (2.3%) | PAROXETINE | 1.4 |
| LEVALBUTEROL | 23 (2.3%) | SIMVASTATIN | 1.4 |
| LEVETIRACETAM | 94 (9.5%) | VITAMIN D | 1.4 |
| LEVOFLOXACIN | 142 (14.3%) | DAPTOMYCIN (CUBICIN) IVPB IN | 1.3 |
| LEVOTHYROXINE | 103 (10.4%) | DORZOLAMIDE | 1.3 |
| LIDOCAINE | 389 (39.3%) | LACOSAMIDE | 1.3 |
| LINEZOLID | 37 (3.7%) | CALCIUM ACETATE | 1.2 |
| LIPASE-PROTEASE-AMYLASE | 8 (0.8%) | CISATRACURIUM | 1.2 |
| LIRAGLUTIDE | 1 (0.1%) | NIMODIPINE | 1.2 |
| LISINOPRIL | 191 (19.3%) | PROCHLORPERAZINE EDISYLATE | 1.2 |
| LITHIUM CARBONATE ER | 2 (0.2%) | RISPERIDONE | 1.2 |
| LOPERAMIDE | 51 (5.1%) | SODIUM FERRIC GLUCONATE | 1.2 |
| LOPINAVIR-RITONAVIR | 1 (0.1%) | SODIUM HYPOCHLORITE | 1.2 |
| LORATADINE | 41 (4.1%) | SOTALOL | 1.2 |
| LORAZEPAM | 303 (30.6%) | VALSARTAN | 1.2 |
| LOSARTAN | 70 (7.1%) | CEFTAZIDIME | 1.1 |
| LOVASTATIN | 7 (0.7%) | CEFUROXIME (ZINACEF) | 1.1 |
| MAFENIDE | 0 (0%) | CHLORTHALIDONE | 1.1 |
| MAGNESIUM CITRATE ORAL SOLUTION | 40 (4%) | DONEPEZIL | 1.1 |
| MAGNESIUM HYDROXIDE | 32 (3.2%) | EZETIMIBE | 1.1 |
| MAGNESIUM OXIDE | 362 (36.5%) | HYDROXYCHLOROQUINE | 1.1 |
| MAGNESIUM SULFATE | 517 (52.2%) | PROTHROMBIN COMPLEX (KCENTRA) INTERMITTENT INFUSION | 1.1 |
| MANNITOL | 19 (1.9%) | BALANCED SALT IRRIGATION SOLUTION (BSS PLAIN) | 1 |
| MATRIX HEMOSTATIC SEALANT | 41 (4.1%) | CALCITRIOL | 1 |
| MEDROXYPROGESTERONE | 1 (0.1%) | CHLORDIAZEPOXIDE | 1 |
| MELATONIN | 203 (20.5%) | CODEINE | 1 |
| MELOXICAM | 1 (0.1%) | DIVALPROEX | 1 |
| MEMANTINE | 6 (0.6%) | GLIPIZIDE | 1 |
| MENTHOL | 28 (2.8%) | POTASSIUM PHOSPHATE, MONOBASIC | 1 |
| MEPERIDINE (DEMEROL) | 17 (1.7%) | PREDNISOLONE ACETATE | 1 |
| MEROPENEM | 82 (8.3%) | TOBRAMYCIN | 1 |
| METFORMIN | 49 (4.9%) | VALGANCICLOVIR | 1 |
| METHADONE | 8 (0.8%) | ACETYLCYSTEINE | 0.9 |
| METHIMAZOLE | 3 (0.3%) | BRIMONIDINE | 0.9 |
| METHOCARBAMOL IVPB IN | 2 (0.2%) | ESOMEPRAZOLE MAGNESIUM DR | 0.9 |
| METHOTREXATE SODIUM | 3 (0.3%) | OXACILLIN | 0.9 |
| METHYLENE BLUE (ANTIDOTE) | 2 (0.2%) | PHENOBARBITAL SODIUM | 0.9 |
| METHYLNALTREXONE | 7 (0.7%) | POTASSIUM & SODIUM PHOSPHATES | 0.9 |
| METHYLPHENIDATE | 1 (0.1%) | PRENATAL VITAMIN WITH CALCIUM NO.72-IRON | 0.9 |
| METHYLPREDNISOLONE | 114 (11.5%) | REMDESIVIR IN NS | 0.9 |
| METOCLOPRAMIDE | 93 (9.4%) | VALPROIC ACID IVPB IN | 0.9 |
| METOLAZONE | 371 (37.4%) | ARIPIPRAZOLE | 0.8 |
| METRONIDAZOLE | 142 (14.3%) | CARBIDOPA | 0.8 |
| MICAFUNGIN IVPB | 25 (2.5%) | CLEVIDIPINE | 0.8 |
| MIDAZOLAM | 380 (38.3%) | DIPHENOXYLATE-ATROPINE | 0.8 |
| MIDODRINE | 21 (2.1%) | DRONABINOL | 0.8 |
| MILRINONE | 2 (0.2%) | FAT EMULSION | 0.8 |
| MINOCYCLINE | 4 (0.4%) | FOSFOMYCIN TROMETHAMINE | 0.8 |
| MIRTAZAPINE | 34 (3.4%) | IMMUNE GLOB,GAMM(IGG) | 0.8 |
| MOMETASONE-FORMOTEROL HFA | 1 (0.1%) | LIPASE-PROTEASE-AMYLASE | 0.8 |
| MONTELUKAST | 31 (3.1%) | METHADONE | 0.8 |
| MORPHINE | 382 (38.5%) | RANOLAZINE ER | 0.8 |
| MOUTHWASH | 332 (33.5%) | ROPINIROLE | 0.8 |
| MOXIFLOXACIN | 5 (0.5%) | TOPIRAMATE | 0.8 |
| MULTIVITAMIN | 206 (20.8%) | AMITRIPTYLINE | 0.7 |
| MUPIROCIN | 88 (8.9%) | DORNASE ALFA | 0.7 |
| MYCOPHENOLATE | 21 (2.1%) | FENOFIBRATE | 0.7 |
| NALOXONE | 22 (2.2%) | GLUCAGON | 0.7 |
| NAPROXEN | 5 (0.5%) | LOVASTATIN | 0.7 |
| NEOMYCIN | 25 (2.5%) | METHYLNALTREXONE | 0.7 |
| NICARDIPINE | 73 (7.4%) | NORTRIPTYLINE | 0.7 |
| NICOTINE | 5 (0.5%) | PSYLLIUM HUSK (ASPARTAME) | 0.7 |
| NIFEDIPINE ER | 14 (1.4%) | SILVER NITRATE | 0.7 |
| NIMODIPINE | 12 (1.2%) | TBO-FILGRASTIM | 0.7 |
| NINTEDANIB | 1 (0.1%) | AZATHIOPRINE | 0.6 |
| NITROGLYCERIN | 120 (12.1%) | BASILIXIMAB INFUSION | 0.6 |
| NITROPRUSSIDE | 3 (0.3%) | CINACALCET | 0.6 |
| NOREPINEPHRINE | 199 (20.1%) | ERTAPENEM (INVANZ) | 0.6 |
| NORTRIPTYLINE | 7 (0.7%) | FLUDROCORTISONE | 0.6 |
| NXSTAGE RFP | 36 (3.6%) | GANCICLOVIR (CYTOVENE) IVPB | 0.6 |
| NYSTATIN | 88 (8.9%) | IRON SUCROSE | 0.6 |
| OCTREOTIDE | 18 (1.8%) | MEMANTINE | 0.6 |
| OLANZAPINE | 56 (5.7%) | PHENAZOPYRIDINE | 0.6 |
| OMEPRAZOLE | 58 (5.9%) | PROCHLORPERAZINE IVPB | 0.6 |
| ONDANSETRON | 422 (42.6%) | RACEPINEPHRINE | 0.6 |
| OSELTAMIVIR | 15 (1.5%) | URSODIOL | 0.6 |
| OSIMERTINIB | 1 (0.1%) | VITAMIN B | 0.6 |
| OXACILLIN | 9 (0.9%) | ADENOSINE | 0.5 |
| OXANDROLONE | 2 (0.2%) | AMMONIUM LACTATE | 0.5 |
| OXCARBAZEPINE | 3 (0.3%) | COLLAGENASE CLOSTRIDIUM HISTOLYTICUM | 0.5 |
| OXYBUTYNIN CHLORIDE | 16 (1.6%) | ESMOLOL | 0.5 |
| OXYCODONE | 393 (39.7%) | FOSPHENYTOIN IV | 0.5 |
| OXYCODONE-ACETAMINOPHEN | 68 (6.9%) | MOXIFLOXACIN | 0.5 |
| OXYMETAZOLINE | 20 (2%) | NAPROXEN | 0.5 |
| PACLITAXEL (TAXOL) IVPB | 1 (0.1%) | NICOTINE | 0.5 |
| PANTOPRAZOLE | 458 (46.2%) | PROTAMINE | 0.5 |
| PAPAVERINE | 37 (3.7%) | SALIVA STIMULANT AGENTS | 0.5 |
| PAROXETINE | 14 (1.4%) | SODIUM ACETATE | 0.5 |
| PEG | 28 (2.8%) | AMANTADINE HCL | 0.4 |
| PENTAMIDINE IVPB IN | 1 (0.1%) | ATOVAQUONE | 0.4 |
| PENTOBARBITAL | 1 (0.1%) | BUPRENORPHINE | 0.4 |
| PERAMIVIR PF (RAPIVAB) IVPB DOSES | 1 (0.1%) | CALCITONIN (SALMON) | 0.4 |
| PERFLUTRE | 62 (6.3%) | CEFTAROLINE IVPB IN | 0.4 |
| PHENAZOPYRIDINE | 6 (0.6%) | CITRATE DEXTROSE SOLUTION | 0.4 |
| PHENOBARBITAL SODIUM | 9 (0.9%) | DOXAZOSIN | 0.4 |
| PHENOL | 35 (3.5%) | FLECAINIDE | 0.4 |
| PHENYLEPHRINE | 130 (13.1%) | GLIMEPIRIDE | 0.4 |
| PHENYTOIN SODIUM EXTENDED | 3 (0.3%) | ISOPROTERENOL INFUSION | 0.4 |
| PHYTONADIONE (VITAMIN K1) | 60 (6.1%) | MINOCYCLINE | 0.4 |
| PIPERACILLIN-TAZOBACTAM | 186 (18.8%) | POSACONAZOLE | 0.4 |
| POLYETHYLENE GLYCOL | 408 (41.2%) | PYRIDOSTIGMINE BROMIDE | 0.4 |
| POSACONAZOLE | 4 (0.4%) | PYRIDOXINE (VITAMIN B6) | 0.4 |
| POTASSIUM & SODIUM PHOSPHATES | 9 (0.9%) | SUMATRIPTAN | 0.4 |
| POTASSIUM CHLORIDE | 639 (64.5%) | TERAZOSIN | 0.4 |
| POTASSIUM CITRATE ER | 2 (0.2%) | VORICONAZOLE | 0.4 |
| POTASSIUM PHOSPHATE IVPB | 92 (9.3%) | AZELASTINE | 0.3 |
| POTASSIUM PHOSPHATE, MONOBASIC | 10 (1%) | BELLADONNA ALKALOIDS-OPIUM | 0.3 |
| POTASSIUM, SODIUM PHOSPHATES | 81 (8.2%) | BROMOCRIPTINE | 0.3 |
| PRAMIPEXOLE | 2 (0.2%) | CARBOPLATIN (PARAPLATIN) IVPB (BY AUC) | 0.3 |
| PRASUGREL | 21 (2.1%) | CLOBETASOL | 0.3 |
| PRAVASTATIN | 72 (7.3%) | CYCLOSPORINE | 0.3 |
| PREDNISOLONE ACETATE | 10 (1%) | ENALAPRIL MALEATE | 0.3 |
| PREDNISOLONE SODIUM PHOSPHATE | 2 (0.2%) | EXPAREL ADMINISTERED WITHIN | 0.3 |
| PREDNISONE | 112 (11.3%) | FLUOROMETHOLONE | 0.3 |
| PREGABALIN | 48 (4.8%) | FORMOTEROL FUMARATE | 0.3 |
| PRENATAL VITAMIN WITH CALCIUM NO.72-IRON | 9 (0.9%) | LAMOTRIGINE | 0.3 |
| PROCHLORPERAZINE EDISYLATE | 12 (1.2%) | METHIMAZOLE | 0.3 |
| PROCHLORPERAZINE IVPB | 6 (0.6%) | METHOTREXATE SODIUM | 0.3 |
| PROCHLORPERAZINE MALEATE | 17 (1.7%) | NITROPRUSSIDE | 0.3 |
| PROMETHAZINE | 111 (11.2%) | OXCARBAZEPINE | 0.3 |
| PROPOFOL | 284 (28.7%) | PHENYTOIN SODIUM EXTENDED | 0.3 |
| PROPRANOLOL | 27 (2.7%) | RASBURICASE (ELITEK) IN | 0.3 |
| PROTAMINE | 5 (0.5%) | RIZATRIPTAN | 0.3 |
| PROTHROMBIN COMPLEX (KCENTRA) INTERMITTENT INFUSION | 11 (1.1%) | SUCRALFATE-LIDOCAINE | 0.3 |
| PSYLLIUM HUSK (ASPARTAME) | 7 (0.7%) | THYROID (PORK) | 0.3 |
| PYRIDOSTIGMINE BROMIDE | 4 (0.4%) | BANANA BAG | 0.2 |
| PYRIDOXINE (VITAMIN B6) | 4 (0.4%) | BENZOCAINE | 0.2 |
| QUETIAPINE | 50 (5%) | BICALUTAMIDE | 0.2 |
| RACEPINEPHRINE | 6 (0.6%) | BIVALIRUDIN | 0.2 |
| RALTEGRAVIR | 1 (0.1%) | BRENTUXIMAB VEDOTIN (ADCETRIS) IVPB | 0.2 |
| RANOLAZINE ER | 8 (0.8%) | CALCIUM CITRATE-VITAMIN D3 | 0.2 |
| RASBURICASE (ELITEK) IN | 3 (0.3%) | CHLORPROMAZINE | 0.2 |
| REMDESIVIR IN NS | 9 (0.9%) | CILOSTAZOL | 0.2 |
| RIFAMPIN | 2 (0.2%) | CONJUGATED ESTROGENS | 0.2 |
| RIFAXIMIN | 17 (1.7%) | DIBUCAINE | 0.2 |
| RISPERIDONE | 12 (1.2%) | ECONAZOLE | 0.2 |
| RIVAROXABAN | 21 (2.1%) | ESTRADIOL | 0.2 |
| RIZATRIPTAN | 3 (0.3%) | FACTOR VIIA RECOMB (NOVOSEVEN) INTERMITTENT INFUSION | 0.2 |
| ROCURONIUM | 27 (2.7%) | FOSAPREPITANT (EMEND) IVPB | 0.2 |
| ROPINIROLE | 8 (0.8%) | IOPAMIDOL | 0.2 |
| ROSUVASTATIN | 1 (0.1%) | LITHIUM CARBONATE ER | 0.2 |
| SALIVA STIMULANT AGENTS | 5 (0.5%) | METHOCARBAMOL IVPB IN | 0.2 |
| SENNOSIDES | 235 (23.7%) | METHYLENE BLUE (ANTIDOTE) | 0.2 |
| SERTRALINE | 41 (4.1%) | MILRINONE | 0.2 |
| SEVELAMER | 26 (2.6%) | OXANDROLONE | 0.2 |
| SILVER NITRATE | 7 (0.7%) | POTASSIUM CITRATE ER | 0.2 |
| SILVER SULFADIAZINE | 55 (5.5%) | PRAMIPEXOLE | 0.2 |
| SIMETHICONE | 36 (3.6%) | PREDNISOLONE SODIUM PHOSPHATE | 0.2 |
| SIMVASTATIN | 14 (1.4%) | RIFAMPIN | 0.2 |
| SMOG ENEMA | 18 (1.8%) | TAMOXIFEN | 0.2 |
| SODIUM ACETATE | 5 (0.5%) | TETANUS-DIPHTHERIA TOXOIDS-TD | 0.2 |
| SODIUM BICARBONATE | 153 (15.4%) | TEZACAFTOR | 0.2 |
| SODIUM CHLORIDE | 790 (79.7%) | THEOPHYLLINE | 0.2 |
| SODIUM FERRIC GLUCONATE | 12 (1.2%) | TRIAMTERENE | 0.2 |
| SODIUM HYPOCHLORITE | 12 (1.2%) | VITAMIN A | 0.2 |
| SODIUM PHOSPHATES | 51 (5.1%) | AMPHOTERICIN B LIPOSOMAL (AMBISOME) IVPB IN | 0.1 |
| SODIUM POLYSTYRENE SULFONATE | 20 (2%) | ANAKINRA | 0.1 |
| SOTALOL | 12 (1.2%) | ARGATROBAN | 0.1 |
| SPIRONOLACTONE | 47 (4.7%) | BENDAMUSTINE (BENDEKA) IVPB | 0.1 |
| STUDY 2330 PENTOBARBITAL | 1 (0.1%) | BENZOIN-ALOE VERA-STORAX-TOLU BALSAM | 0.1 |
| STUDY A-TREAT TRANEXAMIC ACID/PLACEBO TABLET | 1 (0.1%) | BENZTROPINE | 0.1 |
| SUCCINYLCHOLINE CHLORIDE | 28 (2.8%) | BIOTIN | 0.1 |
| SUCRALFATE | 30 (3%) | BUTAMBEN-TETRACAINE-BENZOCAINE | 0.1 |
| SUCRALFATE-LIDOCAINE | 3 (0.3%) | CARTEOLOL | 0.1 |
| SUGAMMADEX | 1 (0.1%) | CHOLESTYRAMINE-ASPARTAME | 0.1 |
| SULFAMETHOXAZOLE | 29 (2.9%) | CLADRIBINE (LEUSTATIN) IVPB | 0.1 |
| SUMATRIPTAN | 4 (0.4%) | CLOBAZAM | 0.1 |
| TACROLIMUS | 16 (1.6%) | CYCLOSPORINE MODIFIED | 0.1 |
| TAMOXIFEN | 2 (0.2%) | CYTARABINE (ARA-C) IVPB | 0.1 |
| TAMSULOSIN | 91 (9.2%) | DANTROLENE | 0.1 |
| TBO-FILGRASTIM | 7 (0.7%) | DAUNORUBICIN (CERUBIDINE) SYRINGE | 0.1 |
| TEDUGLUTIDE | 1 (0.1%) | DIATRIZOATE MEGLUMINE-DIATRIZOATE SODIUM | 0.1 |
| TERAZOSIN | 4 (0.4%) | DIPYRIDAMOLE | 0.1 |
| TETANUS-DIPHTHERIA TOXOIDS-TD | 2 (0.2%) | DONOR LEVOTHYROXINE | 0.1 |
| TEZACAFTOR | 2 (0.2%) | DROXIDOPA | 0.1 |
| THEOPHYLLINE | 2 (0.2%) | DUTASTERIDE | 0.1 |
| THIAMINE HCL | 140 (14.1%) | EMOLLIENT COMBO NUMBER | 0.1 |
| THROMBIN | 94 (9.5%) | EMTRICITABINE-TENOFOVIR | 0.1 |
| THYROID (PORK) | 3 (0.3%) | ETHACRYNATE SODIUM | 0.1 |
| TICAGRELOR | 32 (3.2%) | ETHACRYNIC ACID | 0.1 |
| TIOTROPIUM BROMIDE | 38 (3.8%) | FONDAPARINUX | 0.1 |
| TOBRAMYCIN | 10 (1%) | GLUCOSE | 0.1 |
| TOCILIZUMAB (ACTEMRA) IVPB IN | 1 (0.1%) | HYDROXYUREA | 0.1 |
| TOPIRAMATE | 8 (0.8%) | INDOMETHACIN | 0.1 |
| TORSEMIDE | 15 (1.5%) | LACTASE | 0.1 |
| TRAMADOL | 150 (15.1%) | LANTHANUM | 0.1 |
| TRAZODONE | 72 (7.3%) | LIRAGLUTIDE | 0.1 |
| TRIAMCINOLONE | 16 (1.6%) | LOPINAVIR-RITONAVIR | 0.1 |
| TRIAMTERENE | 2 (0.2%) | MEDROXYPROGESTERONE | 0.1 |
| TUBERCULIN PPD | 15 (1.5%) | MELOXICAM | 0.1 |
| URSODIOL | 6 (0.6%) | METHYLPHENIDATE | 0.1 |
| VALACYCLOVIR | 25 (2.5%) | MOMETASONE-FORMOTEROL HFA | 0.1 |
| VALGANCICLOVIR | 10 (1%) | NINTEDANIB | 0.1 |
| VALPROIC ACID IVPB IN | 9 (0.9%) | OSIMERTINIB | 0.1 |
| VALSARTAN | 12 (1.2%) | PACLITAXEL (TAXOL) IVPB | 0.1 |
| VANCOMYCIN | 455 (45.9%) | PENTAMIDINE IVPB IN | 0.1 |
| VASOPRESSIN | 117 (11.8%) | PENTOBARBITAL | 0.1 |
| VECURONIUM | 34 (3.4%) | PERAMIVIR PF (RAPIVAB) IVPB DOSES | 0.1 |
| VENLAFAXINE | 17 (1.7%) | RALTEGRAVIR | 0.1 |
| VERAPAMIL | 64 (6.5%) | ROSUVASTATIN | 0.1 |
| VITAMIN A | 2 (0.2%) | STUDY 2330 PENTOBARBITAL | 0.1 |
| VITAMIN B | 6 (0.6%) | STUDY A-TREAT TRANEXAMIC ACID/PLACEBO TABLET | 0.1 |
| VITAMIN D | 14 (1.4%) | SUGAMMADEX | 0.1 |
| VORICONAZOLE | 4 (0.4%) | TEDUGLUTIDE | 0.1 |
| WARFARIN | 39 (3.9%) | TOCILIZUMAB (ACTEMRA) IVPB IN | 0.1 |
| ZINC SULFATE | 45 (4.5%) | ZIPRASIDONE | 0.1 |
| ZIPRASIDONE | 1 (0.1%) | ALVIMOPAN | 0 |
| ZOLPIDEM | 22 (2.2%) | MAFENIDE | 0 |

**Supplementary Table 4.** Mean medication cluster distribution for each patient cluster.

|  | **Patient Cluster 1  (n = 234)** | **Patient Cluster 2  (n = 201)** | **Patient Cluster 3  (n = 115)** | **Patient**  **Cluster 4  (n = 247)** | **Patient**  **Cluster 5  (n= 194)** |
| --- | --- | --- | --- | --- | --- |
| Medication Cluster 1 | 0.117 | 0.077 | 0.145 | 0.046 | 0.052 |
| Medication Cluster 2 | 0.072 | 0.064 | 0.074 | 0.052 | 0.063 |
| Medication Cluster 3 | 0.073 | 0.063 | 0.116 | 0.052 | 0.083 |
| Medication Cluster 4 | 0.083 | 0.068 | 0.137 | 0.054 | 0.12 |
| Medication Cluster 5 | 0.045 | 0.033 | 0.039 | 0.017 | 0.035 |
| Medication Cluster 6 | 0.61 | 0.695 | 0.488 | 0.779 | 0.647 |

**Supplementary Table 5.** Permutation MANOVA test based on Bray–Curtis dissimilarity and centered data.

|  | DF | Sum of squares | F statistic | p-value |
| --- | --- | --- | --- | --- |
| Patient cluster | 4 | 25.16 | 20.375 | 0.001 |
| Residual | 986 | 304.34 |  |  |
| Total | 990 | 329.5 |  |  |

**Supplementary Figure 1.** Plot of explained variance versus principal components to determine optimal number of principal components for PCA


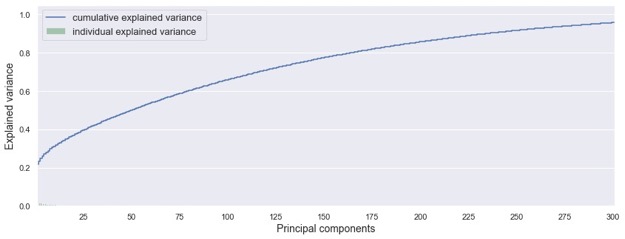


**Supplemental Figure 2.** Silhouette plot for patient clusters


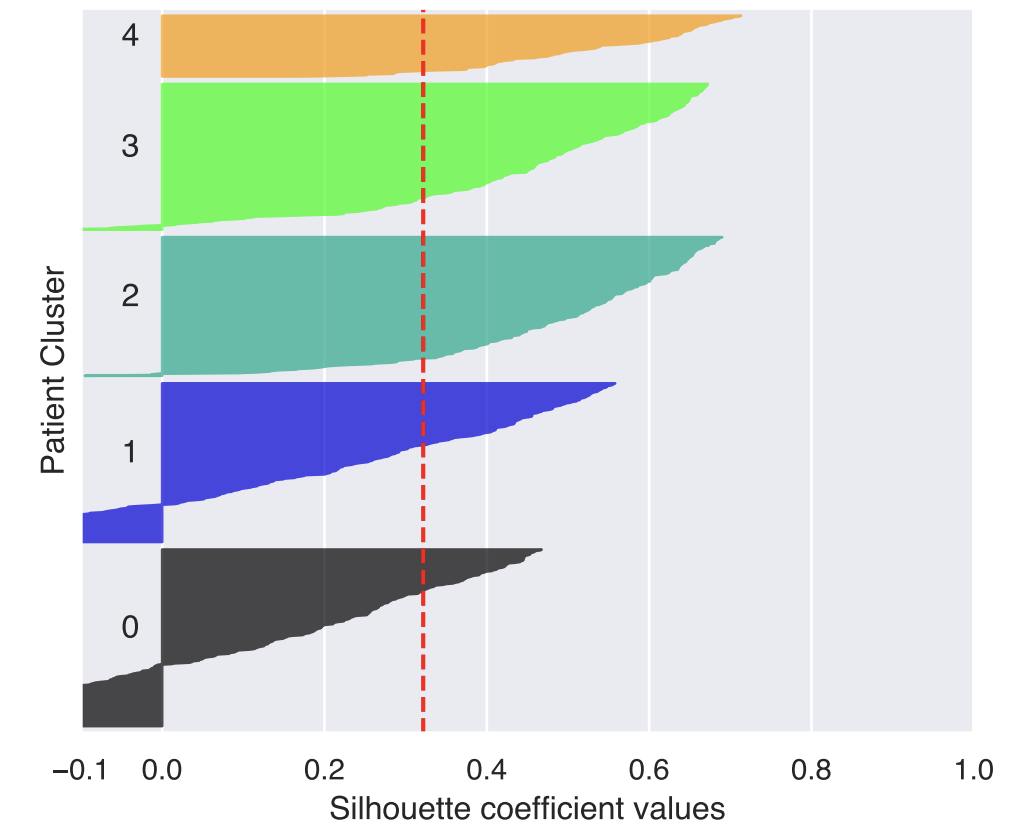


**Supplementary Figure 3.** Box plot showing distribution of clinical outcome per patient cluster without removal of outliers.


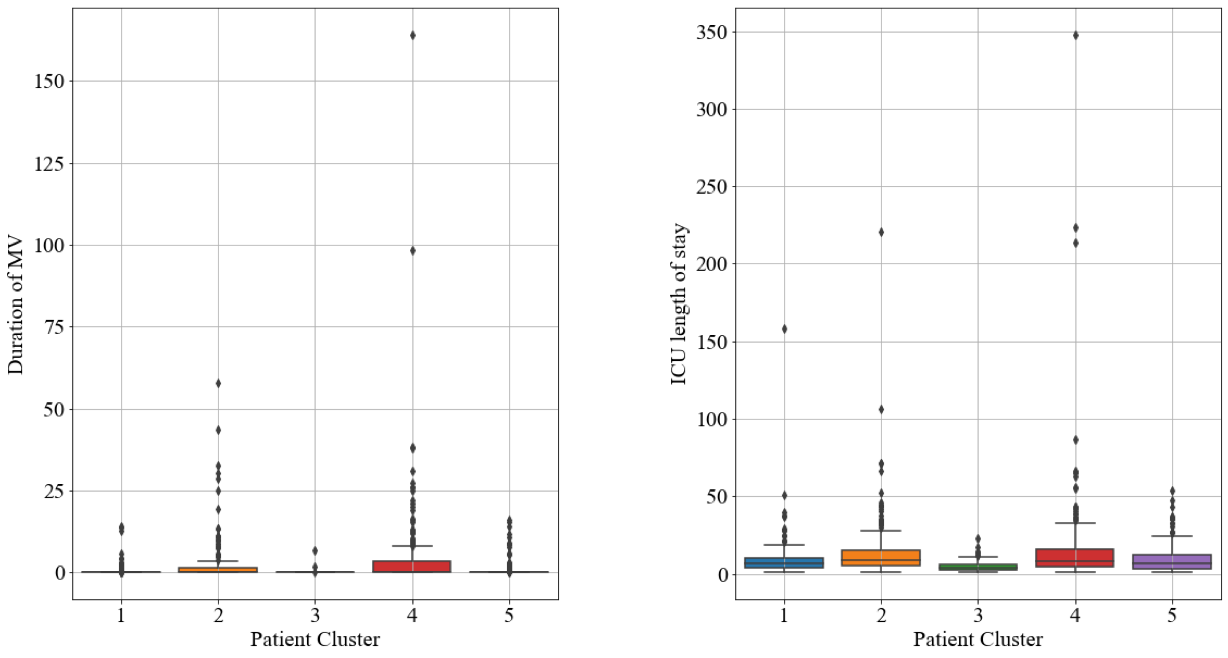

Supplement: Supplementary file 1 — Supplementary Information. [file 41598_2023_42657_MOESM1_ESM.docx]
